# Drought-Induced Stress Priming in Two Distinct Filamentous Saprotrophic Fungi

**DOI:** 10.1007/s00248-019-01481-w

**Published:** 2020-01-16

**Authors:** Alexander Guhr, Sophia Kircher

**Affiliations:** Department of Soil Ecology, BayCEER, University of Bayreuth, Dr.-Hans-Frisch-Straße 1-3, 95448 Bayreuth, Germany

**Keywords:** Drought stress, Saprotrophic filamentous fungi, Stress memory, Stress priming

## Abstract

Sessile organisms constantly face environmental fluctuations and especially drought is a common stressor. One adaptive mechanism is “stress priming,” the ability to cope with a severe stress (“triggering”) by retaining information from a previous mild stress event (“priming”). While plants have been extensively investigated for drought-induced stress priming, no information is available for saprotrophic filamentous fungi, which are highly important for nutrient cycles. Here, we investigated the potential for drought-induced stress priming of one strain each of two ubiquitous species, *Neurospora crassa* and *Penicillium chrysogenum*. A batch experiment with 4 treatments was conducted on a sandy soil: exposure to priming and/or triggering as well as non-stressed controls. A priming stress was caused by desiccation to pF 4. The samples were then rewetted and after 1-, 7-, or 14-days of recovery triggered (pF 6). After triggering, fungal biomass, respiration, and β-glucosidase activity were quantified. *P. chrysogenum* showed positive stress priming effects. After 1 day of recovery, biomass as well as β-glucosidase activity and respiration were 0.5 to 5 times higher during triggering. Effects on biomass and activity decreased with prolonged recovery but lasted for 7 days and minor effects were still detectable after 14 days. Without triggering, stress priming had a temporary negative impact on biomass but this reversed after 14 days. For *N. crassa*, no stress priming effect was observed on the tested variables. The potential for drought-induced stress priming seems to be species specific with potentially high impact on composition and activity of fungal communities considering the expected increase of drought events.

**Electronic supplementary material:**

The online version of this article (10.1007/s00248-019-01481-w) contains supplementary material, which is available to authorized users.

## Introduction

Sessile organisms like fungi have to deal with the temporal dynamics of their environment. These include fluctuations in humidity, temperature, and osmolarity. One way of adapting to environmental stress is by retaining information from previous encounters with stress events. Such priming systems seem to be widely distributed and can also be found in organisms (e.g., plants or microorganisms) without a dedicated nervous system or an adaptive immune system [1, 2]. This process is termed “stress priming” and describes a phenotypic plasticity of response traits without an underlying change of the genome [3]. Mild stress events (“priming”) can lead to an induction of basal defense mechanisms, which can improve resistance to a more severe stress event (“triggering”) following an intermediate recovery phase. The stress priming system is based on molecular and physiological changes in response to environmental stress [4–6].

The potential for stress priming has been studied in a wide range of plant species [7–9]. By contrast, only a few studies are available for microorganisms, e.g., for *Escherichia coli* and *Saccharomyces cerevisiae* [10–12]. Stress priming has the potential to increase survival rates of microorganisms by up to 10 times compared with non-priming conditions [13]. However, the induction of defense mechanisms can be accompanied by a metabolic cost due to a redirection of resources otherwise used for growth or reproduction with potentially negative impact in the absence of a subsequent triggering stress [14]. Hence, it can also potentially affect the microbial community composition if only a part of the community is susceptible to stress priming or if the metabolic cost of the stress priming responses varies among species [13, 15]. Yet, investment in stress priming responses by weak competitors might pay off even at high metabolic costs and provide a chance to outcompete otherwise strong competitors following a triggering [15].

Studies on stress priming of saprotrophic filamentous fungi are only sparsely available, although they play an outstanding role for the nutrient cycle and transport in soils [16–18] and can contribute significantly to the soil microbial biomass [19]. Recently, Andrade-Linares et al. [20] found a positive impact of temperature priming on the performance of some soil filamentous fungi under heat stress but the impact decreased at prolonged recovery times. Stress priming by drought of filamentous saprotrophic soil fungi has to our knowledge not been studied so far. However, drought is one of the major environmental stressors negatively affecting organisms as well as agriculture worldwide and soil water potentials strongly impact biological processes, nutrient cycling, and microbial communities [14, 21–23]. Thus, knowledge of stress priming of saprotrophic filamentous fungi is of great interest for predicting microbial impact on soil biogeochemistry with regard to an expected increasing number of drought events.

Here, we investigated the effect of a mild drought stress event (pF 4), after a recovery of 1-, 3-, or 7- days, on the biomass and activity under a severe triggering stress (pF 6) of one strain each of two filamentous fungi, *Neurospora crassa* and *Penicillium chrysogenum*. We conducted a laboratory batch experiment using an artificial sandy soil. We hypothesized that stress priming by drought (1) stimulates fungal biomass and activity under a severe stress event and (2) that the effect of stress priming decreases with a prolonged recovery time.

## Materials and Methods

### Experimental Design

Stress priming experiments were carried out with single fungal species setups of *Penicillium chrysogenum* Thom (DSM No.: 21171) and *Neurospora crassa* Shear & Dodge (DSM No.: 1259) in glass Petri dishes with a 200-mm diameter. Both species are commonly and ubiquitously found in soils, belong to the best studied filamentous fungi species, including complete available genomes, and show fast growth rates [24, 25]. Further, they strongly vary in their tolerance to drought with *P. chrysogenum* showing a substantially higher xerotolerance than *N. crassa* [26, 27].

Petri dishes were filled as a 10-mm layer with a homogenized and steam sterilized mixture of mineral soil (2:1:1 v/v/v mixture of loamy soil (17% clay; 76% silt; 7% sand), medium coarse quartz sand (Dorsilit 8, particle size range 0.3–0.8 mm) and coarse quartz sand (Dorsilit 7, 0.6–1.2 mm, both Dorfner GmbH & Co., Hirschau, Germany). The artificial soil was inoculated by placing a 1-cm^2^ agar plate (malt extract peptone agar) with fungal hyphae on top of the soil. As the soil was nearly nutrient free, all samples were adjusted one-time to a water content equivalent to 50% of the maximum water holding capacity (WHC) with a liquid growth medium (2% glucose, 0.2% peptone, 0.2% yeast extract, 0.1% K_2_HPO_4_, 0.46% KH_2_PO_4_, and 0.05% MgSO_4_; [28]). Subsequently, samples were pre-incubated for approximately 3 weeks in a climate chamber at 20 °C until fungal growth was observable all over the soil surface.

**Fig. 1:**
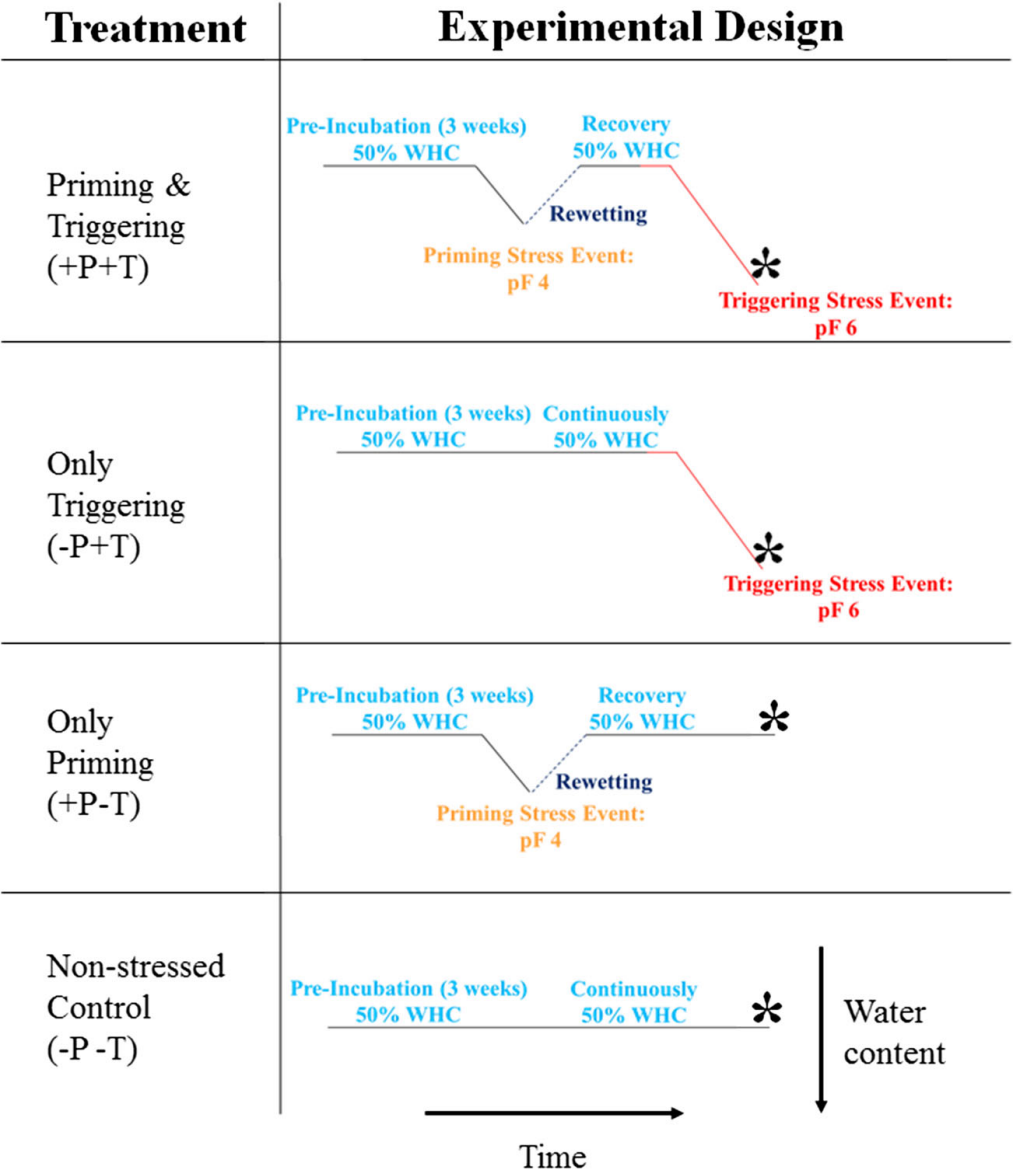
Overview of the experimental procedure. In total, 4 treatments were applied: samples were pre-incubated at 50% water holding capacity (WHC) prior to exposure to a priming stress event (desiccation to pF 4) and/or a triggering stress event (desiccation to pF 6) with an intermediary recovery phase (1-, 7-, or 14-days) as well as non-stressed controls. Asterisks indicate the sampling time for the measurement of fungal biomass, respiration, and β-glucosidase activity when pF 6 was reached in the triggered samples

At the end of the pre-incubation period under standardized conditions, the experiment was started by randomly opening half of the Petri dishes. The samples were first desiccated for 1 day until a pF value of 4 was reached “priming.” Subsequently, the samples were slowly adjusted again to 50% WHC with sterilized tap water to avoid differences in nutrient contents among treatments and the lids were kept closed for either 1-, 7-, or 14-days “recovery phase”. After the recovery phase, the lids were opened again for 3 days until a water potential of − 100 MPa (pF 6, “triggering”, treatment is referred to as “+P+T”) was reached. In addition, a non-primed control group of Petri dishes was directly subjected to the triggering stress without a prior priming stress and recovery (referred to as “−P+T”). Furthermore, another group of Petri dishes was exposed to the priming stress and rewetted to 50% WHC but not exposed to a subsequent triggering stress (referred to as “+P−T”). Finally, a further control group of Petri dishes was neither exposed to a priming nor a triggering stress but maintained at 50% WHC throughout the experiment (referred to as “−P−T”, for a graphical overview of the treatments, see Fig. 1). All analyses were done in 4 replicates and at each recovery time, 4 new independent Petri dishes per treatment were sampled. Petri dishes were only sampled if no contaminations were visible.

### Analytical Methods

Four Petri dishes per group and recovery time were destructively harvested after pF 6 was reached in the triggered treatments. Fungal biomass carbon was measured by the chloroform-fumigation extraction method [29]. Subsamples of 8 g were extracted with 0.5 M K_2_SO_4_ with a soil:solution ratio of 1:5 [30]. The total dissolved carbon analyses in the solutions were done with a TOC-V CPN analyzer (Shimadzu, Kyōto, Japan).

To measure the soil respiration rate, subsamples of 10 g were placed into a 50-mL air-tight crimp vial. The CO_2_ production of the substrates was measured by gas chromatography (GC 8610C, SRI Instruments, Torrance, USA) after 0 and 24 h. CO_2_ production rate was calculated by the linear increase in CO_2_ concentration during the 24 h incubation, corrected for actual air pressure and air temperature.

The impact of stress priming on the soil enzyme activity at the triggering stress was done using soil zymography [31–33]. This allowed the analyses of enzyme activities under different water contents in contrast to more traditional methods based on the determination of enzyme activity in solution. The β-glucosidase activity was analyzed using the artificial substrate 4-methylumbelliferyl β-d-glucopyranoside (4-MG, Sigma-Aldrich Chemie GmbH, Schnelldorf, Germany). The fluorogenic 4-methylumbelliferone (MUF) is released from 4-MG due to hydrolytic cleavage in presence of compatible enzymes. Polyamide membrane filters were saturated with 4-MG and placed on the soil surface (filters: stripes of 5 × 10 cm). The membrane filters were extracted after 30 min at 20 °C and illuminated on a fluorescent transilluminator in the dark (wavelength 355 nm, Desaga GmbH, Wiesloch, Germany) and mean values of the grayscale were measured with imageJ 1.52a (Wayne Rasband, National Institutes of Health, USA, 2014). Enzyme activates were calculated based on a calibration line of different MUF concentrations (membranes of 2 cm^2^ with 0, 35, 70, 130, 200 μM; measured as described above) as μg MUF released per mm^2^ soil area within 1 h. Values were standardized based on the difference between a control filter slice (no incubation on the soil and photographed with the others) and the calibration membrane with 0 μM MUF concentration to adjust for differences in exposure time.

### Data Analyses

All statistical analyses and graphics were done using R 3.5.1 [34]. Normality and homogeneity of the data were tested using Shapiro-Wilk test and Levene’s test, respectively. Significant differences between groups were tested by *t* test or Mann-Whitney *U* test independently for each recovery time. Only pairwise comparisons between primed and non-primed samples, independently for either triggered (“+P+T” vs. “−P+T”) or non-triggered conditions (“+P−T” vs. “−P−T”), were done as the strong difference in soil water content would overshadow all potential differences resulting from the priming. Therefore, we calculated the ln-transformed response ratio (lnR) to facilitate the comparison of effect sizes and enable a comparison of triggered and non-triggered samples: lnR = ln(*E*/*C*), where *E* is the measured value of the response variable in the primed treatments and *C* is the mean value of the response variable in the controls [35, 36].

## Results

**Fig. 2:**
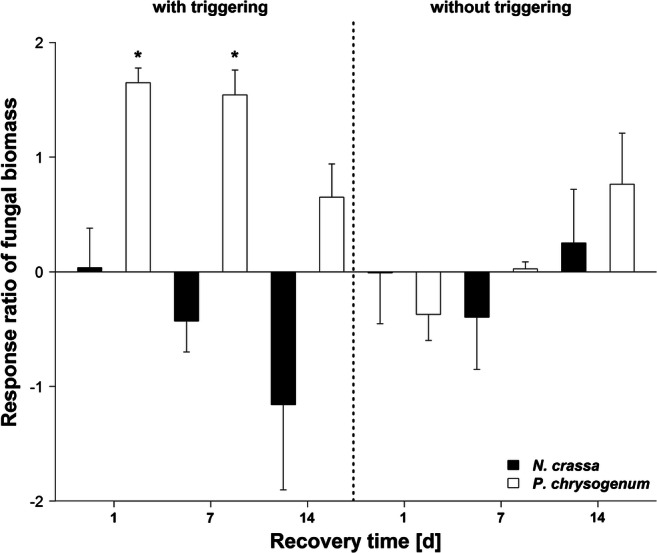
Changes in fungal biomass carbon in response to priming (pF4) of *Penicillium chrysogenum* and *Neurospora crassa* with and without a subsequent triggering event (pF6) in comparison with non-primed samples (ln-transformed response ratio). Asterisks indicate significant differences between primed and non-primed samples of *P. chrysogenum* or *N. crassa* tested independently for each recovery time and independent for triggered (“+P+T” vs. “−P+T”) and non-triggered (“+P−T” vs. “−P−T”) conditions (*p* < 0.05). Mean ± SE, *n* = 4

In the case of *P. chrysogenum*, fungal biomass was about 5 times higher if the samples were primed before a triggering stress (+P+T) and 1 or 7 days of recovery were applied compared with the non-primed samples (−P+T; *p* < 0.05, Fig. 2, Table S1). Even after 14 days of recovery, fungal biomass was still on average 2 times but not significantly higher in the primed samples (+P+T) compared with the non-primed samples (−P+T; *p* > 0.05). Without a subsequent triggering, fungal biomass was on average halved after priming (+P−T) and 1 day of recovery compared with the non-stressed controls (−P−T). With prolonged recovery, biomass recovered and was on average even about 2 times higher in the primed samples (+P−T) compared with the non-stressed controls (−P−T) after 14 days of recovery. For *N. crassa*, priming seemed to have a generally negative impact on fungal biomass but no clear and significant trends were detectable (*p* > 0.05).

**Fig. 3:**
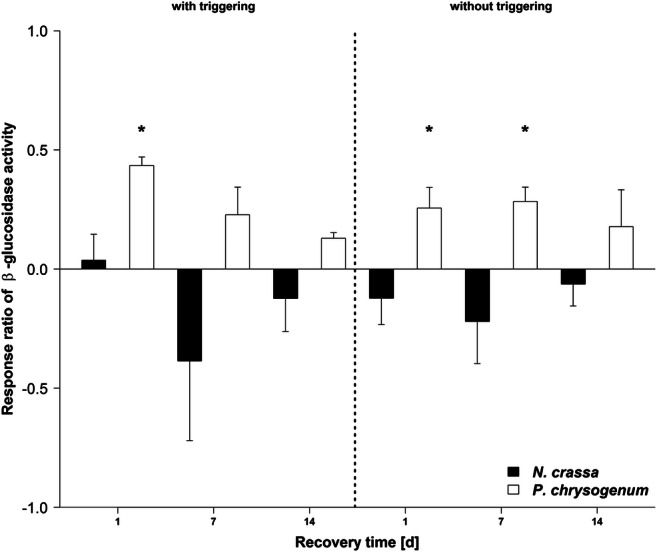
Changes in β-glucosidase activity in response to priming (pF4) of *Penicillium chrysogenum* and *Neurospora crassa* with and without a subsequent triggering event (pF6) in comparison with non-primed samples (ln-transformed response ratio). Asterisks indicate significant differences between primed and non-primed samples of *P. chrysogenum* or *N. crassa* tested independently for each recovery time and independent for triggered (“+P+T” vs. “−P+T”) and non-triggered (“+P−T” vs. “−P−T”) conditions (*p* < 0.05). Mean ± SE, *n* = 4

Similar trends were observed for β-glucosidase activity (Fig. 3, Table S1). After 1 day recovery, β-glucosidase activities were about 0.5 times higher if *P. chrysogenum* was primed before the triggering (+P+T) compared with the non-primed samples (−P+T; *p* < 0.05). With prolonged recovery times, β-glucosidase activities were still on average higher in primed samples (+P+T) compared with the non-primed samples (−P+T) but the differences regressed with time (*p* > 0.05). Without a subsequent triggering, β-glucosidase activities were in all cases on average and in 2 cases significantly higher after priming (+P−T; 1 and 14 days of recovery, *p* < 0.05) compared with the non-stressed controls (−P−T). In *N. crassa*, priming seemed to have again a generally negative impact but no significant differences were detected (*p* > 0.05).

**Fig. 4:**
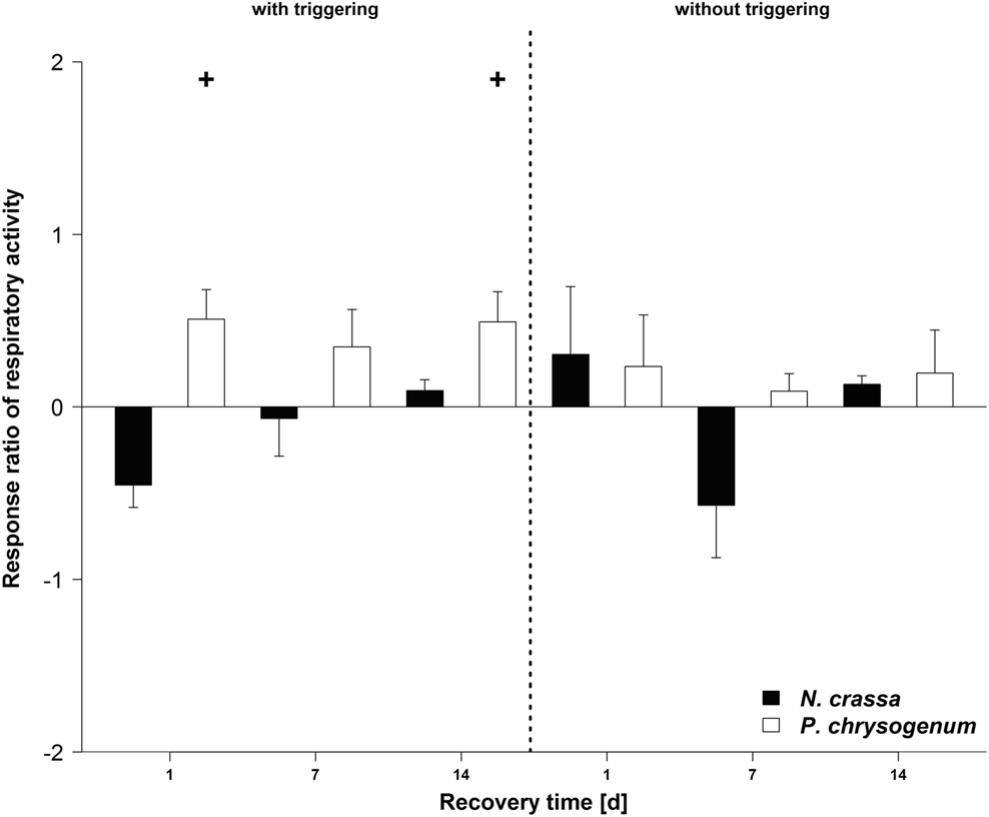
Changes in respiratory activity in response to priming (pF4) of *Penicillium chrysogenum* and *Neurospora crassa* with and without a subsequent triggering event (pF6) in comparison with non-primed samples (ln-transformed response ratio). Daggers indicate a tendency for a statistical difference between primed and non-primed samples of *P. chrysogenum* or *N. crassa* tested independently for each recovery time and independent for triggered (“+P+T” vs. “−P+T”) and non-triggered (“+P−T” vs. “−P−T”) conditions (*p* < 0.1). Mean ± SE, *n* = 4

Respiratory activity was on average 0.5 times higher if *P. chrysogenum* was primed before the triggering (+P+T) compared with the non-primed samples (−P+T) but there was only a tendency for a statistical effect of priming after 1 and 14 days of recovery with no change over time (*p* < 0.1, Fig. 4, Table S1). Without triggering of *P. chrysogenum* as well as for *N. crassa* with and without triggering, no clear trends were observable (*p* > 0.05).

## Discussion

Here, we show for the first time the potential of drought-induced stress priming in a strain of a filamentous fungus. While *P. chrysogenum* showed positive stress priming effects in biomass, in β-glucosidase, and in respiratory activity under a severe drought stress event, no effects were observed for *N. crassa* in any of the tested variables. Variations between both species are likely based on differences in physiological traits (see discussion below).

### Effects of Stress Priming on *P. chrysogenum*

After 1 and 7 days of recovery, fungal biomass, β-glucosidase activity, and respiration of *P. chrysogenum* were substantially higher after stress priming compared with non-primed samples during the triggering. This indicates that *P. chrysogenum* seems to have the potential to maintain information systems of previous encounters with drought stress for at least 7 days leading to an enhanced or faster response after a second exposure [3]. Consequently, survival rates as well as metabolic activity are strongly increased during the subsequent triggering event. This potential for stress priming might be one explanation for the observed relatively high xerotolerance of *P. chrysogenum* [27]. It is also likely one reason for the ubiquitous distribution of *P. chrysogenum*, being one of the most numerous eukaryotes on earth [24].

The positive effect of stress priming seemed to decrease with time yet fungal biomass and β-glucosidase were still on average but not significantly higher after 14 days of recovery. Hence, it can be expected that the observed duration of the stress-primed phase is considerably longer than previously reported for temperature priming in filamentous fungi (up to 12 h) [20]. However, durations of the stress-primed phase in plants were also described up to several days [8] and in some cases even over an entire vegetation period [7]. A similar duration of the stress-primed phase over several days in filamentous fungi is therefore not unexpected.

Stress priming without a subsequent triggering had only minor and variable effects in *P. chrysogenum*. Nevertheless, a few tendencies emerged. The lower biomass of *P. chrysogenum* in response to the priming treatment compared with the non-stressed controls after 1 day of recovery indicates that metabolic costs are associated with the establishment of the primed state [3, 14]. The production of protective compounds and structures as well as the activation of epigenetic regulators is energy consuming and probably accompanied with a shift of resources otherwise used for growth [14]. An increased demand for energy is also indicated by the higher β-glucosidase and respiratory activity in response to the stress priming without triggering compared with the non-stressed controls. Yet, with increasing recovery time, the negative effect seemed to have been attenuated and stress priming seemed to have even stimulated growth after 14 days. An increase of hyphal biomass in response to drought is not uncommon and has been previously documented in field studies [37, 38].

### Effects of Stress Priming on *N. crassa*

For *N. crassa*, no effects of stress priming were detectable at the tested recovery times. In general, the priming stress seemed to have negatively impacted all measured variables but no clear trends were observable. This might be related to the relatively low xerotolerance of *N. crassa* [39]. Loss of membrane permeability as well as inhibition of germination and growth was reported even at minor reductions of the water activity comparable with the applied priming stress [26]. Therefore, the priming stress may have already caused severe damage to the hyphae without potential to redistribute resources to defense mechanisms. Further, species with a high growth rate like *N. crassa* are considered to mostly invest in a short-term duration of the stress-primed phase [3, 40]. A recovery time of 1 day might therefore be already long enough to lose the stored information. Indeed, Kapoor and Sveenivasan [41] reported an induced thermotolerance of *N. crassa* after a previous milder heat shock treatment without any intermediary recovery. Therefore, a shorter recovery time might have led to an increased drought tolerance in this species as well. However, a faster rewetting and desiccation of the soil could have introduced an independent drying/rewetting stress, complicating the interpretation of the results.

### Regulation of Stress Priming Responses and Potential Effects on Fungal Communities

How stress priming responses are regulated on the molecular level is still only partly understood. In general, the stress priming response seems to be regulated by intracellular signaling networks and especially epigenetic mechanisms seem to be important [42–44]. The response is often mediated by mechanisms like histone modifications or the activity of transcription factors, leading to changes at the transcriptional, post-transcriptional, translational, and posttranslational level [45–47]. While histone demethylation seems to be especially important in the regulation of the priming response in plants [48], a recent study indicated that histone acetylation may be the more important regulator in saprotrophic filamentous fungi [49]. While outside the scope of this study, more research on the molecular regulations of stress priming in filamentous fungi is required, i.e., transcriptomic/proteomic approaches or the use of known molecular inhibitors to interfere with the stress priming process.

In any way, stress priming may strongly impact the fungal community composition upon drought events that are predicted to increase in the future. As the response to stress priming seems to be strongly species specific, the potential shift in the microbial community may highly depend on the intensity, frequency, and time lag between drought stress events [13]. For instance, time lags between priming and triggering within the duration of the primed phase probably favor species with the potential for stress priming*.* However, species without a stress priming response may be on advantage if no subsequent triggering occurs. Further, high intensities as well as high frequencies of drought stress events probably shift the fungal community towards species with the potential for stress priming.

## Conclusions

We demonstrate that the potential for drought-induced stress priming can be found in a strain of the saprotrophic filamentous fungus *P. chrysogenum* with positive effects on biomass and activity under a severe stress event. Thereby, the primed phase seems to be potentially active for at least 1 week and even after 14 days of recovery, minor effects were still observable. In addition, stress priming seems to be associated with metabolic costs and negative impact on biomass for a short time in absence of a subsequent triggering event. However, the potential for priming seems to be species specific with potentially high impact on the composition and activity of fungal communities with and without a subsequent triggering event.

## Electronic Supplementary Material

ESM 1 (DOCX 18 kb)
