# Supplementary material for: Drought-Induced Stress Priming in Two Distinct Filamentous Saprotrophic Fungi
Source: Microb Ecol. 2020 Jan 16;80(1):27–33. doi: 10.1007/s00248-019-01481-w (PMC7338827; doi:10.1007/s00248-019-01481-w)
Supplement: Supplementary file 1 — (DOCX 18 kb) [file 248_2019_1481_MOESM1_ESM.docx]

**Supplementary information**

**Drought-induced stress priming in two distinct filamentous saprotrophic soil fungi**

**Authors:** Alexander Guhr^a^ and Sophia Kircher^a^

**Affiliations:**

^a^Department of Soil Ecology, BayCEER, University of Bayreuth, Dr.-Hans-Frisch-Straße 1-3, 95448 Bayreuth

**Corresponding author:**

Alexander Guhr

Email: alexander.guhr@uni-bayreuth.de

**Table S1** Effect of stress priming by drought on microbial biomass, β-glucosidase activity and respiratory activity of *Neurospora crassa* and *Penicillium chrysogenum*. Samples were either exposed to priming (pF 4) and/or triggering (pF6) as well as kept at non-stress conditions. Mean ± SE, n= 4.

| **Species** | **Recovery time [d]** | **Treatment** | **Microbial Biomass [mg C kg^-1^ soil DW]** | **Enzyme activity [pmol mm^-^² h^-1^]** | **Respiratory activity [µg C-CO_2_ kg^-1^ soil DW h^-1^]** |
| --- | --- | --- | --- | --- | --- |
| *P. chrysogenum* | 1 | Non-stressed control (-P –T) | 124.86 ± 15.90 | 13.55 ± 0.34 | 26.56 ± 5.10 |
| *P. chrysogenum* | 1 | only priming (+P –T) | 93.43 ± 22.68 | 17.71 ± 1.53 | 38.30 ± 11.10 |
| *P. chrysogenum* | 1 | only triggering (-P +T) | 29.69 ± 7.87 | 9.70 ± 1.26 | 1.67 ± 0.30 |
| *P. chrysogenum* | 1 | priming and triggering (+P +T) | 158.05 ± 17.84 | 15.01 ± 0.54 | 2.90 ± 0.46 |
| *P. chrysogenum* | 7 | Non-stressed control (-P –T) | 123.83 ± 27.69 | 7.95 ± 0.71 | 48.00 ± 10.35 |
| *P. chrysogenum* | 7 | only priming (+P –T) | 127.79 ± 8.34 | 10.61 ± 0.66 | 53.38 ± 5.49 |
| *P. chrysogenum* | 7 | only triggering (-P +T) | 20.65 ± 8.76 | 6.71 ± 0.64 | 1.89 ± 0.44 |
| *P. chrysogenum* | 7 | priming and triggering (+P +T) | 101.49 ± 20.12 | 8.61 ± 1.09 | 2.88 ± 0.67 |
| *P. chrysogenum* | 14 | Non-stressed control (-P –T) | 74.75 ± 3.16 | 9.05 ± 1.07 | 29.84 ± 2.78 |
| *P. chrysogenum* | 14 | only priming (+P –T) | 198.86 ± 53.76 | 11.17 ± 1.49 | 39.31 ± 7.83 |
| *P. chrysogenum* | 14 | only triggering (-P +T) | 33.6 ± 14.71 | 9.76 ± 1.00 | 3.01 ± 0.29 |
| *P. chrysogenum* | 14 | priming and triggering (+P +T) | 72.91 ± 20.36 | 11.12 ± 0.25 | 5.15 ± 0.87 |
| *N. crassa* | 1 | Non-stressed control (-P –T) | 78.40 ± 14.50 | 12.99 ± 0.54 | 31.36 ± 2.16 |
| *N. crassa* | 1 | only priming (+P –T) | 98.45 ± 32.23 | 11.72 ± 1.39 | 53.9 ± 21.77 |
| *N. crassa* | 1 | only triggering (-P +T) | 77.74 ± 20.50 | 9.57 ± 1.21 | 7.60 ± 3.31 |
| *N. crassa* | 1 | priming and triggering (+P +T) | 94.98 ± 28.93 | 10.11 ± 1.15 | 4.96 ± 0.63 |
| *N. crassa* | 7 | Non-stressed control (-P –T) | 14.87 ± 3.61 | 10.37 ± 1.02 | 28.73 ± 4.44 |
| *N. crassa* | 7 | only priming (+P –T) | 13.65 ± 6.33 | 8.70 ± 1.36 | 19.05 ± 6.95 |
| *N. crassa* | 7 | only triggering (-P +T) | 38.35 ± 7.95 | 7.08 ± 0.34 | 4.76 ± 2.36 |
| *N. crassa* | 7 | priming and triggering (+P +T) | 27.59 ± 6.50 | 5.50 ± 1.28 | 4.77 ± 0.96 |
| *N. crassa* | 14 | Non-stressed control (-P –T) | 78.66 ± 8.90 | 8.20 ± 0.81 | 34.45 ± 2.66 |
| *N. crassa* | 14 | only priming (+P –T) | 137.23 ± 59.77 | 7.80 ± 0.73 | 39.38 ± 1.93 |
| *N. crassa* | 14 | only triggering (-P +T) | 44.73 ± 8.73 | 5.96 ± 0.71 | 4.02 ± 0.65 |
| *N. crassa* | 14 | priming and triggering (+P +T) | 28.77 ± 18.60 | 5.43 ± 0.79 | 4.44 ± 0.28 |
